# Selection and Validation of the Optimal Panel of Reference Genes for RT-qPCR Analysis in the Developing Rat Cartilage

**DOI:** 10.3389/fgene.2020.590124

**Published:** 2020-12-16

**Authors:** Liang Liu, Hui Han, Qingxian Li, Ming Chen, Siqi Zhou, Hui Wang, Liaobin Chen

**Affiliations:** ^1^Department of Orthopedic Surgery, Zhongnan Hospital of Wuhan University, Wuhan, China; ^2^Department of Pharmacology, Wuhan University School of Basic Medical Sciences, Wuhan, China; ^3^Hubei Provincial Key Laboratory of Developmentally Originated Disease, Wuhan, China

**Keywords:** intrauterine growth retardation, real-time quantitative polymerase chain reaction, cartilage development, prenatal dexamethasone exposure, the panel of reference genes

## Abstract

Real-time fluorescence quantitative PCR (RT-qPCR) is widely used to detect gene expression levels, and selection of reference genes is crucial to the accuracy of RT-qPCR results. Minimum Information for Publication of RT-qPCR Experiments (MIQE) proposes that using the panel of reference genes for RT-qPCR is conducive to obtaining accurate experimental results. However, the selection of the panel of reference genes for RT-qPCR in rat developing cartilage has not been well documented. In this study, we selected eight reference genes commonly used in rat cartilage from literature (*GAPDH, ACTB, 18S, GUSB, HPRT1, RPL4, RPL5*, and *SDHA*) as candidates. Then, we screened out the optimal panel of reference genes in female and male rat cartilage of fetus (GD20), juvenile (PW6), and puberty (PW12) in physiology with stability analysis software of genes expression. Finally, we verified the reliability of the selected panel of reference genes with the rat model of intrauterine growth retardation (IUGR) induced by prenatal dexamethasone exposure (PDE). The results showed that the optimal panel of reference genes in cartilage at GD20, PW6, and PW12 in physiology was *RPL4* + *RPL5*, which was consistent with the IUGR model, and there was no significant gender difference. Further, the results of standardizing the target genes showed that *RPL4* + *RPL5* performed smaller intragroup differences than other panels of reference genes or single reference genes. In conclusion, we found that the optimal panel of reference genes in female and male rat developing cartilage was *RPL4* + *RPL5*, and there was no noticeable difference before and after birth.

## Introduction

Real-time fluorescence quantitative polymerase chain reaction (RT-qPCR) has the advantages of high sensitivity, strong specificity, and high throughput, so it is widely used for the detection of gene expression levels (Nolan et al., 2006; Zhang et al., 2019). Reference genes are the relatively constant genes for expression in tissues or cells, which can calibrate the experimental errors and ensure the accuracy of the experimental results. In general, researchers use reference genes for RT-qPCR analysis to quantify the relative expression of the target genes. Therefore, the selection of reference genes directly affects the accuracy of the experimental results (Bustin et al., 2009). Glyceraldehyde 3-phosphate dehydrogenase (*GAPDH*) and actin beta (*ACTB*) are frequently used as reference genes in considerable experiments. However, studies found that there was no generality in reference genes (de Oliveira et al., 2012; Pinto et al., 2012), and their stability varied by tissues, treatments, and developmental stages. Therefore, using a fixed reference gene often fails to accurately reflect the gene expression levels of various tissues, periods, and treatments (Sharan et al., 2015; Arya et al., 2017). So, it is necessary to select appropriate reference genes according to the model and actual situation.

Articular cartilage is an essential component of the human and animal skeletal system. In general, rats mainly go through three developmental stages: fetal period (gestational day 20, GD20), juvenile (postnatal week 6, PW6), and puberty (PW12), each of which has different biological characteristics. As the embryo develops into a complete individual, articular cartilage also continues to develop. At various stages of cartilage development, the ratio of chondrocytes and gene expression levels will change (Cleary et al., 2015). Moreover, the expression levels of reference genes differ in treatments (Al-Sabah et al., 2016). This indicates that the stability of reference genes may also change at different developmental stages of cartilage or when the cartilage is treated differently. Besides, Minimum Information for Publication of RT-qPCR (MIQE) proposes that using a single reference gene for RT-qPCR analysis may lead to inaccurate results, and the rigorous PCR analysis needs to select a stable panel of reference genes, a combination of two or more reference genes, as a reference to relatively quantify the expression of the target genes (Bustin et al., 2009; Huggett et al., 2013). Therefore, the screening and identification of the panel of reference genes that stably express at cartilage developmental stages are the basis and premise for carrying out studies on cartilage development. However, there is no related research on which genes can be used as a panel of reference genes of articular cartilage at different developmental stages.

The stability of reference genes possibly differs in various pathological models. Therefore, it is not only necessary to explore the reference genes that are stable during the development in physiology, but also to verify under representative development-related disease models. Due to the simple operation, high incidence of intrauterine growth retardation (IUGR), and high stability of the model, the adverse environmental exposure during pregnancy has become a common pathological model for researching developmental diseases (Rahman et al., 2017; Sabra et al., 2017; Dessì et al., 2018; Zhang et al., 2018). A large number of studies have shown that a variety of prenatal adverse environmental exposures, such as exogenous exposure, malnutrition, infection, hypoxia, and stress, can increase intrauterine maternal glucocorticoid levels, resulting in a series of offspring developmental programming changes (Lesage et al., 2001; Buss et al., 2012; Pawluski et al., 2012; Cuffe et al., 2014; Correia-Branco et al., 2015; Sedaghat et al., 2015). Our previous studies also confirmed that prenatal caffeine, nicotine, and ethanol exposure could cause fetal rat maternal glucocorticoid overexposure, accompanied by IUGR and multiple organ and tissue (including cartilage) developmental disorders (Chen et al., 2007; Liang et al., 2011; He et al., 2017; Qing-Xian et al., 2020). Therefore, fetal-maternal glucocorticoid overexposure under prenatal adverse environmental exposure can be a common phenomenon. Moreover, the IUGR model induced by prenatal dexamethasone exposure (PDE) is a classic method (Tomaszewska et al., 2012; Conti et al., 2017; Chen et al., 2018; Liu et al., 2018; Xiao et al., 2018; Li et al., 2019; Wu et al., 2019). Dexamethasone, as a synthetic glucocorticoid, is easy to enter fetus through the placenta (Morales et al., 1986), so the IUGR model induced by PDE can well simulate the overexposure of glucocorticoid in prenatal adverse environments during the development of offspring rats. By screening the sable reference genes in developmental cartilage in the PDE model and verifying the reliability of reference genes screened in physiology, it can help to further improve the accuracy of research results related to cartilage development.

In this study, we first screened out the optimal panel of reference genes in rat articular cartilage at different developmental stages (GD20, PW6, and PW12) in physiology. Next, to confirm the stability and accuracy of the panel of reference genes screened above, we used PDE to construct a rat IUGR model of glucocorticoid overexposure during pregnancy and further compared the consistency of the panel of reference genes in this model with physiology. Finally, we verify the reliability of the selected panel of reference genes by detecting the expression of cartilage-specific functional genes in the IUGR model induced by PDE. This study explored the optimal panel of reference genes in the cartilage development period for the first time in physiology and PDE-induced IUGR model, providing a methodological basis for studies related to cartilage development.

## Materials and Methods

### Chemicals and Reagents

Dexamethasone was obtained from the Shuanghe Pharmaceutical Co. (Wuhan, China). A TRIzol kit (CAS No. 5346994) was purchased from Invitrogen Co. (Carlsbad, CA, United States). Isoflurane was obtained from Baxter Healthcare Co. (Deerfield, IL, United States). Reverse transcription and RT-qPCR kits (CAS No. R232) were obtained from Vazyme Biotech Co., Ltd. (Nanjing, China). Power SYBR^®^ Green PCR Master Mix (CAS No. A46109) was obtained from the Thermo Fisher Scientific Co., Ltd. (Shanghai, China). All primers were synthesized by Tianyi Huiyuan Biotech Co., Ltd. (Wuhan, China). The other chemicals and agents were analytical grade.

### Animals and Treatments

All experimental Specific pathogen-free Wistar rats [No. 2017–0018, license number: SCXK (Hubei), certification number: 42000600002258] with females weighing 201 ± 20 g and males weighing 270 ± 20 g were obtained from Hubei Provincial Center for Disease Control and Prevention (Hubei, China). All of the experimental animal procedures were conducted in accordance with the Guidelines for the Care and Use of Laboratory Animals of the Chinese Animal Welfare Committee. This study was approved by the Animal Experimental Ethics Committee of Wuhan University Medical College (license number: 14016).

The animals were kept in rat cages in an air-conditioned room under standard conditions (room temperature: 18–22°C; humidity: 40–60%; light cycle: 12 h light and dark; and 10–12 air changes per hour) and allowed free access to food and water. All rats were adaptively fed one week before experimentation, and then two female rats were placed together with one male rat overnight in a cage for mating. The vaginal discharge smear of the caged female rat was examined by a microscope the next morning, and if sperm was found, it was confirmed as GD0, as previously described (Dong et al., 2018). Pregnant rats were randomly divided into two groups after confirmation of successful conception: the control and the PDE group. From GD9 to GD20, pregnant rats in the PDE group were injected with dexamethasone (0.2 mg/kg⋅d) subcutaneously at 9 am every day, and the control group was given the same volume of saline.

At GD20, some of the pregnant rats were anesthetized with 2% isoflurane, the fetuses were removed by laparotomy under the ultra-clean platform, and eight pregnant rats with 8 to 14 offspring per litter were included in the experiment. Meanwhile, we recorded the weight and gender of the fetuses. The lower limbs of fetal rats were severed on ice and stored in an ultra-low temperature refrigerator at −80°C for extraction of the total RNA in cartilage. Subsequently, in the postnatal rat experiment, the number of female offspring in each litter was uniformly adjusted to 10 with half males and half females on the first day after birth when the pregnant rats naturally delivered, and each group accommodated eight litters. After weaning at PW4, one female and male rat were selected from each litter and placed into one group and continued to feed to juvenile (PW6), and puberty (PW12). Each group consisted of 8 rats of each sex from 8 litters (*n* = 8). All animals were anesthetized with 2% isoflurane at each time point, decapitated and sacrificed, and the entire lower limb was retained for extraction of the total RNA in cartilage.

### Total RNA Extraction and cDNA Synthesis

The total RNA of cartilage tissue was extracted by TRIzol method following the manufacturer’s instructions. Briefly, after the cartilage (one sample came from one rat, and each group contained 8 samples, i.e., *n* = 8) was obtained under the ultra-clean platform and ground cartilage with a grinder (No. KZ-II, Servicebio), they were transferred into the 1.5 mL EP tube, and added 1 ml TRIzol and 200 μL chloroform. Then the sample was placed for 10 min on ice after fully mixed. After low temperature high speed centrifugation (Centrifuge 5430R, Eppendorf, Germany) at 12000 × *g* and 4°C for 15 min, the clarified liquid (400 μL) of the upper layer was transferred into a new EP tube, and placed for 10 min at room temperature after the same amount of isopropanol was added and mixed. The white precipitates containing total RNA were obtained after centrifugation at 12000 × *g* and 4°C for 10 min. Then washed it with 1 mL of precooled 75% ethanol twice and dissolved it with free RNA enzyme water. The obtained total RNA was treated with DNase to remove DNA pollution after an RNA integrity test with agarose gel electrophoresis showed that it was eligible (Supplementary Figure 1). We used NanoDrop 2000 micronuclei acid analyzer (NanoDrop 2000, Thermo Scientific, United States) to detect the concentration and purity of total RNA to ensure that the A260/A280 ratio of all samples was between 1.8 and 2.0. And we synthesized cDNA according to the instructions of HiScript III RT SuperMix for qPCR (+gDNA wiper) reagent kit. Briefly, after adding 4 μL 4 × gDNA wiper Mix to 1 μg RNA, added RNAase-free ddH_2_O to 16 μL. After mixing, put it into thermal cycler (Veriti Thermal Cycler): 42°C for 2 min; then added 4 μL 5 × HiScript III RT SuperMix at 37°C for 15 min and 85°C for 5 s. Finally, stored it at −20°C after dilution 1: 4.

### Reference Genes Selection, Primers Design, and RT-qPCR

As shown in Supplementary Table 1, we selected eight commonly used reference genes (*GAPDH, ACTB, 18S, HPRT1, RPL4, SDHA, GUSB*, and *RPL5*) in cartilage as candidates by consulting the literature (Pombo-Suarez et al., 2008; Zhai et al., 2013; Zhou et al., 2014; Ma et al., 2015; Al-Sabah et al., 2016; He et al., 2018). We searched the complete cDNA sequence of the reference genes from NCBI’s Entrez Nucleotides database. Primer Premier 5.0 (PREMIER Biosoft International) was used for primer design, and then the designed primers were input into the BLAST database for homology comparison. Finally, the specific primer sequences were obtained (Supplementary Table 2). Next, RT-qPCR was used to detect the expression levels of the selected reference genes of rats cartilage tissues at GD20, PW6, and PW12 in each group. The RT-qPCR reaction was performed using the 2^–ΔΔCt^ method according to the AceQ Universal SYBR qPCR Master Mix reagent instructions. The 10 μL reaction system contained 0.2 μL forward and reversed primer, 1 μL cDNA, 3.6 μL ddH_2_O and 5 μL SYBR. The PCR amplifications were performed using a 7500 Real-Time PCR System (Applied Biosystems, Foster City, CA, United States). The amplification curve reaction program: 50°C for 2 min, 95°C for 10 min, 95°C for 15 s, 60°C for 1 min, 40 cycles; Melting curve at 95°C for 15 min, 60°C for 30 s, and 95°C for 15 min. Finally, we collected the fluorescence signal and drew the standard curve and melting curve. We repeated the tests for three times each sample.

### Standard Curve and Amplification Efficiency

The cDNA product of each sample was divided into eight gradients by four times, and each gradient was set to three replicates. Using the diluted product as the standard, we determined the standard curve of each candidate reference gene. The primer amplification efficiency (E) is related to the slope of the standard curve. According to the slope of the standard curve, the primers amplification efficiency was calculated from the equation $E\left(\%\right)=\left(10^{-\frac{1}{s\,l\,o\,p\,e}}-1\right)\times 100$.

### Software and Statistical Analysis

geNorm, NormFinder, and BestKeeper are three commonly used reference gene stability screening software developed by different teams, and they’re based on different algorithms. GeNorm and NormFinder were, respectively, developed by Vandesompele et al. (2002) and Andersen et al. (2004), and both of them can judge the stability by calculating the stability value (M) of the reference gene. The higher the *M* value, the more unstable the reference gene, and vice versa. If *M* ≤ 1.5, it can be considered that the gene is stably expressed in this tissue and can be used as a reference. GeNorm can calculate the paired variation value *V* of the standardization factor after the introduction of a new reference gene to decide the number of the optimal panel of reference genes, so it can screen the most stable combination of two or more reference genes to correct the data, while NormFinder can only screen the most stable one. When Vn/Vn + 1 < K (K is generally 0.15, but it may be adjusted according to the actual situation), the number of the best panel of reference genes is n. BestKeeper is a gene expression stability analysis software developed by Michael in 2004 for reference genes and target genes by calculating the pairing coefficient (*r*), standard deviation (SD) and coefficient of variation (CV) produced by the pairing between each gene (Pfaffl et al., 2004). The larger the r is, the smaller the SD and CV are, and the more stable the internal reference genes are (Al-Sabah et al., 2016; Lü et al., 2018; Sarker et al., 2018). The expression level of target genes can be analyzed at the same time in BestKeeper software.

Before the experiment, the minimum animal sample size was calculated as 8 based on a power efficiency β = 0.8 and α = 0.05 for detecting a statistical difference between groups under the maximum failure load. And we used SPSS 20 (SPSS Science Inc., Chicago, IL, United States) and Prism 7 (Graph Pad Software, La Jolla, CA, United States) for data statistics and graphing. Quantitative data were expressed as the mean ± S.E.M. An unpaired two-tailed Student’s *t*-test was used for comparisons between control and the PDE group. A one-way analysis of variance (ANOVA) was used in BestKeeper to compare the stability of reference genes. *P* < 0.05 was considered statistically significant for all of the tests.

## Results

### The Specificity and Amplification Efficiency of the Candidate Genes’ Primers

Before the analysis of reference genes’ stability, all the candidate genes’ primers were detected for melting and standard curves. The results showed that the melting curves of all candidate reference genes’ primers displayed a single peak, and the Tm values ranged from 80 to 90°C (Supplementary Figures 2A–H), indicating that the specificity of the selected primers performed well. The *R*^2^ ≥ 0.995 of the standard curves of all candidate reference genes indicated a clear linear relationship between the template cDNA content and the Ct value. According to the slope and *R*^2^ of the standard curves, the candidate reference gene primers’ amplification efficiencies were calculated as 97% ∼ 105% (Supplementary Table 3), which were between 90 ∼ 110% recommended by MIQE (Taylor et al., 2010). Therefore, all primers meet the requirements for further experiments.

### The Expression Levels and Stability Analysis of the Candidate Reference Genes in Rat Cartilage at Various Developmental Stages in Physiology

**FIGURE 1:**
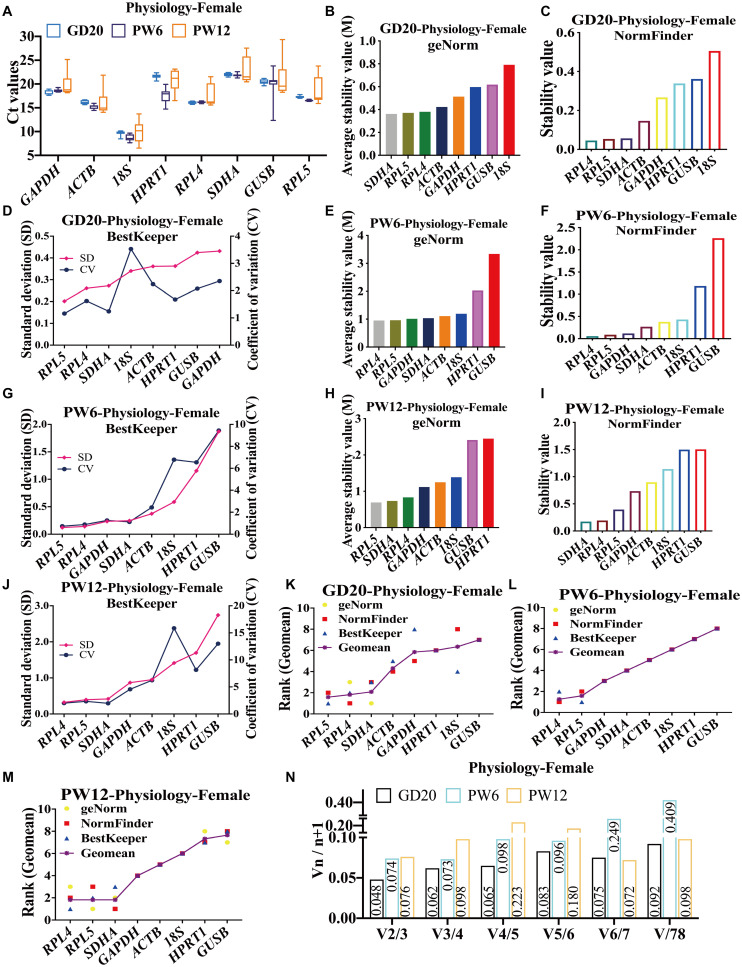
The candidate reference genes’ expression and stability of female rat cartilage in different physiological developmental stages. **(A)** Distribution of Ct values of candidate reference genes in physiological cartilage of female rat at GD20, PW6 and PW12. GeNorm, NormFinder and BestKeeper analyzed the stability of the candidate reference genes in rat cartilage at GD20 **(B–D)**, PW6 **(E–G)** and PW12 **(H–J)** (from most stable to least stable). The comprehensive stability ranking of the candidate reference genes in rat cartilage at GD20 **(K)**, PW6 **(L)**, and PW12 **(M)** decreased from left to right. **(N)** Paired coefficient of variation (CV) of standardization factor’s variation after introducing a new reference gene in rat cartilage under physiological conditions at GD20, PW6, and PW12. *n* = 8.

To screen the stable reference genes in cartilage at developmental stages in physiology, we used RT-qPCR to detect the expression of the above eight reference genes in cartilage of female rats at the three time-points (GD20, PW6, and PW12), and analyzed their stability. The results showed that the Ct values of candidates in female rat articular cartilage were between 8 and 23 at GD20, PW6 and PW12 (Figure 1A). At GD20 and PW12, geNorm, NormFinder, and BestKeeper stability analysis showed that *RPL4*, *RPL5*, and *SDHA* were the most stable reference genes, while *GUSB* and *18S* were the most unstable reference genes (Figures 1B–D, H–J). At PW6, the three software unanimously showed that *RPL4* and *RPL5* had the highest stability, but *GAPDH* ranked third, while *GUSB* was also the most unstable (Figures 1E–G).

In order to further determine the comprehensive stability of the candidate reference genes at various developmental stages (GD20, PW6, and PW12) in female rat cartilage, we calculated the geometric mean of the above three software for ranking of each reference gene. The results showed that under physiological conditions at GD20, the comprehensive gene ranking of the most stable to least stable genes in male rat cartilage was *RPL5, RPL4, SDHA, ACTB, GAPDH, HPRT1, 18S, GUSB* (Figure 1K). At PW6, it was *RPL4, RPL5, GAPDH, SDHA, ACTB, 18S, HPRT1, GUSB* (Figure 1L). And at PW12, the ranking was *RPL4, RPL5, SDHA, ACTB, 18S, HPRT1, GUSB* (Figure 1M). GeNorm further calculated the paired coefficients of standardization factor’s variation after introducing a new reference gene for the three periods and showed that all V2/3 were less than 0.15 (Figure 1N), indicating that the number of the optimal panel of reference genes in female rat cartilage under physiological conditions was two. Therefore, *RPL4* and *RPL5* could be used as the optimal panel of reference genes in female rats’ cartilage at the development stages under physiological conditions.

**FIGURE 2:**
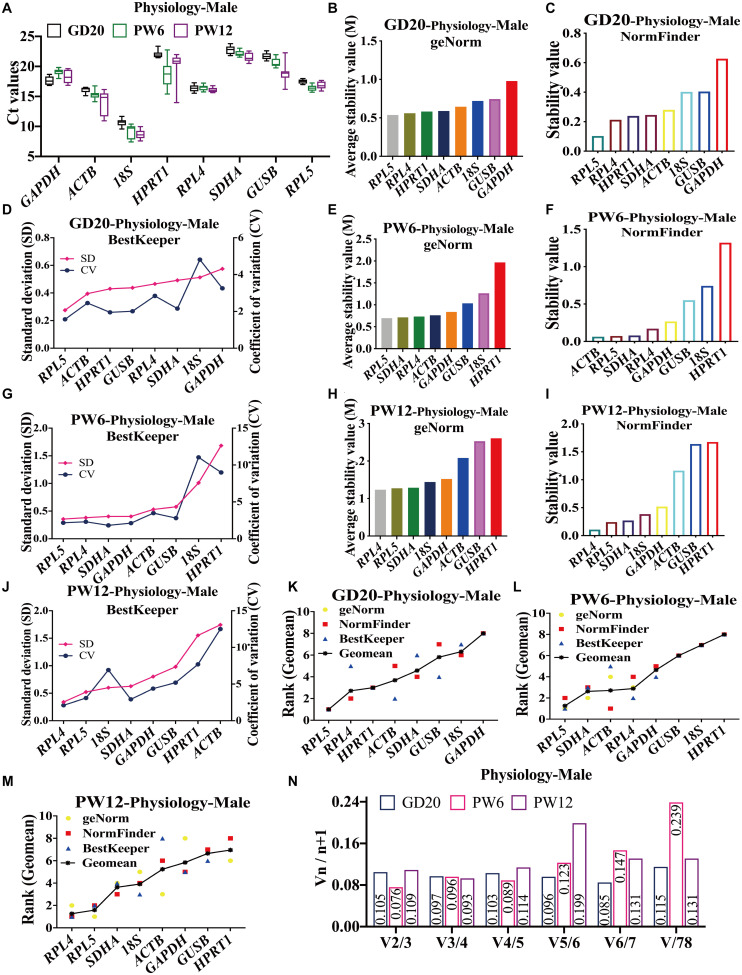
The candidate reference genes’ expression and stability of male rat cartilage in different physiological developmental stages. **(A)** Distribution of Ct values of the candidate reference genes in physiological cartilage of male rat at GD20, PW6, and PW12. GeNorm, NormFinder and BestKeeper analyzed the stability of the candidate reference genes in male rat cartilage at GD20 **(B–D)**, PW6 **(E–G)**, and PW12 **(H–J)** (from most stable to least stable). The comprehensive stability ranking of the candidate reference genes in male rat cartilage at GD20 **(K)**, PW6 **(L)**, and PW12 **(M)** decreased from left to right. **(N)** Paired coefficient of variation (CV) of standardization factor’s variation after introducing a new reference gene in female rat cartilage under physiological conditions at GD20, PW6, and PW12. *n* = 8.

In male rats, the Ct values of all the candidate reference genes ranged from 8 to 23 at GD20, PW6 and PW12 (Figure 2A). At GD20, geNorm and NormFinder showed that *RPL4* and *RPL5* were the most stable reference genes, and *GUSB* and *18S* were the most unstable (Figures 2B,C). BestKeeper showed that *RPL5* and *ACTB* were the top two, and RPL4 was the fifth (Figure 2D). At PW6, geNorm and BestKeeper showed that the stability of *RPL4*, *RPL5*, and *SDHA* ranked in the top three (Figures 2E,G). However, the NormFinder showed that *ACTB* had the highest stability, and *RPL5*, *SDHA*, and *RPL4* were ranked 2 to 4 (Figure 2F). Moreover, all believed that *18S* and *HPRT1* had the worst stability (Figures 2E–G). At PW12, geNorm, NormFinder and BestKeeper all showed that *RPL4* and *RPL5* were also the most stable reference genes (Figures 2H–J); geNorm and NormFinder showed that *GUSB* and *HPRT1* had the worst stability (Figures 2H,I), while BestKeeper thought it was *ACTB* (Figure 2J).

Furthermore, under the physiological conditions at GD20, the comprehensive gene ranking in male rat cartilage was *RPL5, RPL4, HPRT1, ACTB, SDHA, GUSB, 18S, GAPDH* (Figure 2K). At PW6, the ranking was *RPL5, SDHA, ACTB, RPL4, GAPDH, GUSB, 18S, HPRT1* (Figure 2L). And at PW12, it was *RPL4, RPL5, SDHA, 18S, ACTB, GAPDH, GUSB, HPRT1* (Figure 2M). The same as females, *RPL4* and *RPL5* still had the highest stability at GD20 and PW12. However, at PW6, *RPL5* had the highest stability, while RP*L4* ranked fourth. But *RPL4* was also suitable for being a reference gene for its stability value M < 1.5. V2/3 was also less than 0.15 (Figure 2N), which indicated that the number of the optimal panel of reference genes in male rat cartilage under physiological conditions was also two. In summary, *RPL4* and *RPL5* were still the optimal panel of reference genes in male rat cartilage in physiology.

### The Expression Levels and Stability Analysis of the Candidate Reference Genes in Rat Cartilage at Various Developmental Stages in the PDE-Induced IUGR Model

**FIGURE 3:**
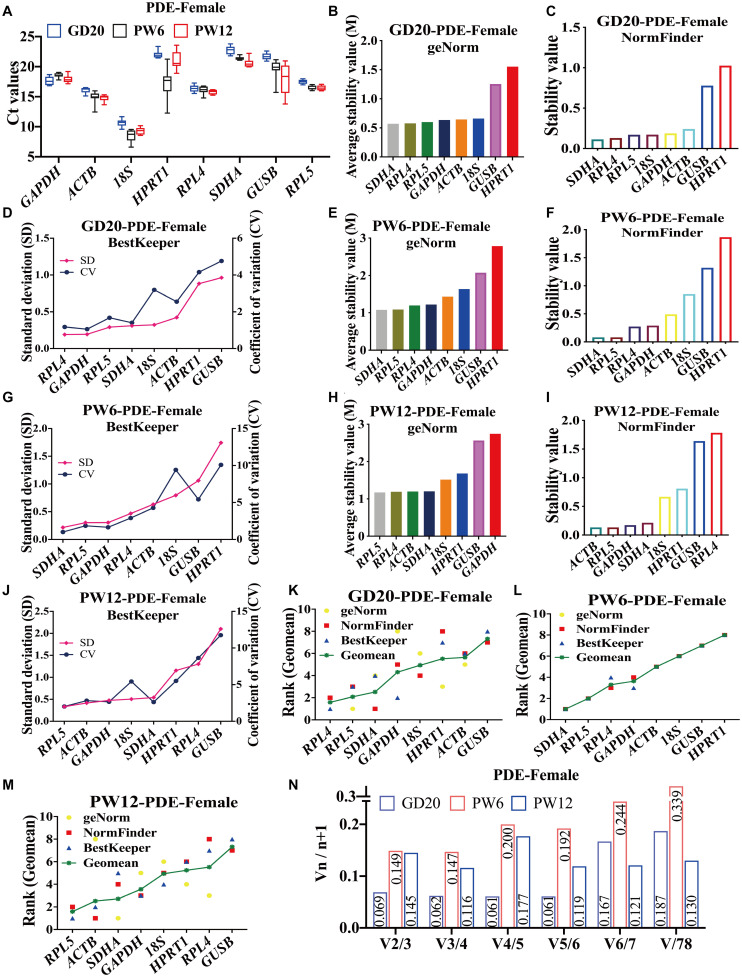
The candidate reference genes’ expression and stability of female rat cartilage in different developmental stages in the PDE-induced IUGR model. **(A)** Distribution of Ct values of the candidate reference genes in female rat cartilage of PDE model at GD20, PW6, and PW12. GeNorm, NormFinder and BestKeeper analyzed the stability of the candidate reference genes in female rat cartilage of PDE model at GD20 **(B–D)**, PW6 **(E–G)**, and PW12 **(H–J)** (from most stable to least stable). The comprehensive stability ranking of the candidate reference genes in female rat cartilage at GD20 **(K)**, PW6 **(L)**, and PW12 **(M)** decreased from left to right. **(N)** Paired coefficient of variation (CV) of standardization factor’s variation after introducing a new reference gene in female rat cartilage under PDE model at GD20, PW6, and PW12. *n* = 8.

To further verify the stability of reference genes screened under pathological conditions, we selected the PDE-induced IUGR rat model for similar analysis. The results showed that in female rats in the PDE-induced IUGR model, the average Ct values of the eight candidate genes were between 8 and 23 at GD20, PW6 and PW12 (Figure 3A). At GD20, both geNorm and NormFinder indicated that *RPL4*, *RPL5*, and *SDHA* were the most stable reference genes (Figures 3B,C), and BestKeeper showed that the stability of *RPL4*, *GAPDH*, and *RPL5* ranked in the top three (Figure 3D), while all the three software showed that *GUSB* and *HPRT1* had the worst stability (Figures 3B–D). At PW6, geNorm and NormFinder also believed that *SDHA*, *RPL4*, and *RPL5* were ranked top three for stability (Figures 3E,F), while BestKeeper thought that *SDHA*, *RPL5*, and *GAPDH* were ranked top three, and *RPL4* was ranked fourth (Figure 3G). *GUSB* and *HPRT1* were also the most unstable at PW6 (Figures 3E–G). At PW12, geNorm showed that the stability of *RPL5* and *RPL4* were also the top two (Figure 3H). But NormFinder and BestKeeper believed that the stability of *ACTB* and *RPL5* were the top two, while *RPL4* ranked 8th and 7th (Figures 3I,J). The different results from the three software may attribute to the algorithm.

At GD20, the comprehensive gene ranking in male rat cartilage in the PDE-induced IUGR model was *RPL4, RPL5, SDHA, GAPDH, 18S, HPRT1, ACTB, GUSB* (Figure 3K); at PW6, the ranking was *SDHA, RPL5, RPL4, GAPDH, ACTB, 18S, GUSB, HPRT1* (Figure 3L); and at PW12, it was *RPL5, ACTB, SDHA, GAPDH, 18S, HPRT1, RPL4, GUSB* (Figure 3M). GeNorm calculated that V2/3 was less than 0.15 (Figure 3N). In summary, at GD20 and PW6, *RPL4* and *RPL5* were also the best panel of reference genes. However, at PW12, due to the differences in the algorithms of the three software, the results of the comprehensive ranking were different. If only the geNorm ranking was considered at PW6, *RPL4* and *RPL5* could still be used as the best panel of reference genes in male rat cartilage in the PDE-induced IUGR model.

**FIGURE 4:**
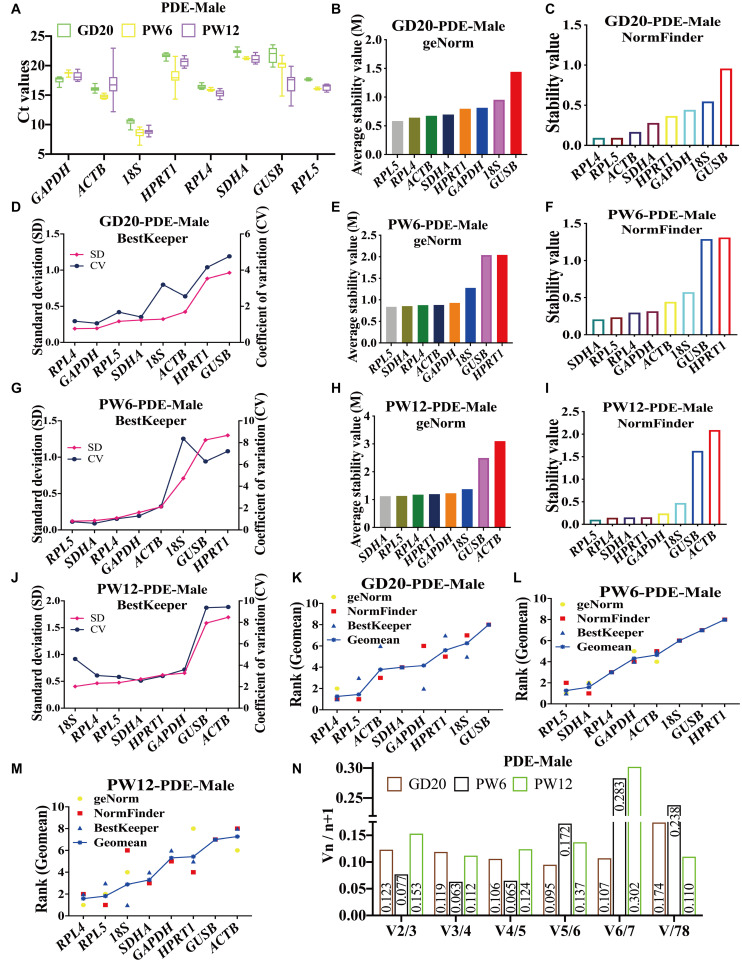
The candidate reference genes’ expression and stability of male rat cartilage in different developmental stages in the PDE model. **(A)**: Distribution of Ct values of the candidate reference genes in cartilage at GD20, PW6 and PW12. GeNorm, NormFinder and BestKeeper analyzed the stability of the candidate reference genes in male rat cartilage at GD20 **(B–D)**, PW6 **(E–G)** and PW12 **(H–J)** (from most stable to least stable). The comprehensive stability ranking of the candidate reference genes in the male rat cartilage in PDE model at GD20 **(K)**, PW6 **(L)**, and PW12 **(M)** decreased from left to right. **(N)** Paired coefficient of variation (CV) of standardization factor’s variation after introducing a new reference gene in the male rat cartilage in PDE model at GD20, PW6, and PW12. *n* = 8.

In male rat cartilage of the PDE-induced IUGR model, all the candidate reference genes showed Ct values also ranged from 8 to 23 at GD20, PW6, and PW12 (Figure 4A). At GD20, the stability analysis of geNorm and NormFinder showed that *RPL4* and *RPL5* were first and second, respectively, and *18S* and *GUSB* had the worst stability (Figures 4B,C). BestKeeper believed that the stability of *RPL4* and *GAPDH* was in the top two, and *RPL5* was ranked third (Figure 4D). At PW6, although the ranking was different, geNorm, NormFinder, and BestKeeper all showed that the stability of *RPL4*, *RPL5*, and *SDHA* was still in the top three. At the same time, *GUSB* and *HPRT1* were the most unstable (Figures 4E–G). At PW12, both geNorm and NormFinder believed that the stability of *SDHA* and *RPL5* was the top two (Figures 4H,I), but BestKeeper considered *18S* and *RPL4* as the top two, while *RPL5* and *SDHA*, respectively ranked third and fourth (Figure 4J). And all three software considered *ACTB* to be the most unstable (Figures 4H–J).

In the PDE-induced IUGR model, at GD20, the comprehensive ranking in male rat cartilage was *RPL4, RPL5, ACTB, SDHA, GAPDH, HPRT1, 18S, GUSB* (Figure 4K); at PW6, it was *RPL5, SDHA, RPL4, GAPDH, ACTB, 18S, GUSB, HPRT1* (Figure 4L); at PW12, it was *RPL4, RPL5, 18S, SDHA, GAPDH, HPRT1, GUSB, ACTB* (Figure 4M). The same as female rats, geNorm calculated that V2/3 in all three periods was also less than 0.15 (Figure 4N). In summary, at GD20 and PW12, *RPL4* and *RPL5* had the highest stability. While at PW6, *RPL5*, and *SDHA* had the highest stability, and *RPL4* ranked third. If the stability of the reference gene and the simplicity and economy of the experiment were considered comprehensively, *RPL4* and *RPL5* could still be selected as the panel of reference genes at PW6. As a result, *RPL4* and *RPL5* were always the optimal panel of reference genes in male rats under the PDE-induced IUGR model.

### Verification of Stability and Accuracy of Cartilage Reference Genes in the PDE-Induced IUGR Model

**FIGURE 5:**
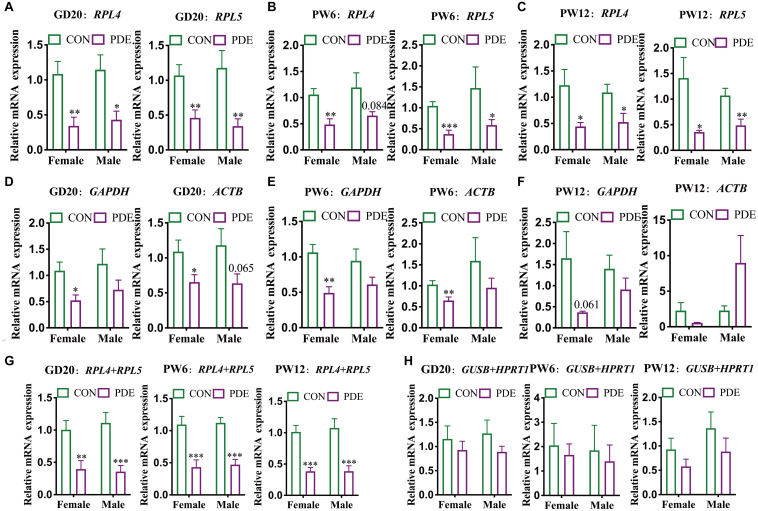
Different reference genes normalized ACAN mRNA expression in cartilage in different developmental periods. RPL4 and RPL5 normalized ACAN mRNA expression in cartilage at GD20 **(A)**, PW6 **(B)**, and PW12 **(C)**. GAPDH and ACTB normalized ACAN mRNA expression in cartilage at GD20 **(D)**, PW6 **(E)**, and PW12 **(F)**. RPL4 + RPL5 **(G)** and GUSB + HPRT1 **(H)**, respectively, normalized ACAN mRNA expression in cartilage at GD20, PW6, and PW12. The values were the means ± S.E.M, *n* = 8. ^∗^*P* < 0.05, ^∗∗^*P* < 0.01, ^∗∗∗^*P* < 0.001 vs. control.

To further verify the stability and reliability of the screened panel of reference genes, we used the PDE-induced IUGR rat model to confirm the influence of using a single-reference gene or panel of reference genes on the mRNA expression by standardizing cartilage functional gene *ACAN* at GD20, PW6, and PW12. A large number of early animal and cell experiments had shown that the matrix content of cartilage in each of the above periods decreased significantly in the PDE-induced IUGR model, and the expression levels of *ACAN* mRNA and protein were also considerably reduced (Iwaniak et al., 2016; Chen et al., 2018; Xiao et al., 2020). The verification results showed that when normalized with the two single reference genes screened above (*RPL4* and *RPL5*), the mRNA expression level of *ACAN* was significantly reduced in cartilage of male and female rats in the above three periods (*P* = 0.084, *P* < 0.05, *P* < 0.01; Figures 5A–C). It should be noted that the mRNA expression of *ACAN* in rat cartilage standardized by *SDHA* was also significantly reduced (*P* = 0.069, *P* < 0.05, *P* < 0.01; Supplementary Figure 3A). However, except for the decrease of *ACAN* mRNA expression in female rats cartilage standardized by *GAPDH* and *ACTB* (*P* < 0.05, *P* < 0.01; Figures 5D,E), there was no significant change in the expression of *ACAN* mRNA when normalized with other single reference genes (*ACTB*, *18S, HPRT1, GAPDH*, and *GUSB*) (Figures 5D–F and Supplementary Figure 3B). It showed that the target gene expression could also show statistical differences when *GAPDH* and *ACTB* were used for standardization, but *RPL4* and *RPL5* had higher stability and reliability than *GAPDH* and *ACTB*.

Besides, we further verified the mRNA expression levels of ACAN with the panel of reference genes. When the two reference genes (*RLP4* + *RPL5*) screened above were used as the panel of reference genes, the mRNA expression levels of *ACAN* in the cartilage of male and female rats in all the three stages were significantly reduced (*P* < 0.01, *P* < 0.001; Figure 5G). Because of the relative stability of *SDHA* indicated from the above results, we also verified that when *RPL4* + *SDHA* and *RPL5* + *SDHA* were used as the panel of reference genes, the mRNA expression levels of *ACAN* in the cartilage of male and female rats in the three periods were also significantly reduced (*P* < 0.01, *P* < 0.001; Supplementary Figures 4A–C). When other panels of reference genes (*GUSB* + *HPRT1*; *RPL4* + *HPRT1*) were selected, there were no significant changes except for the reduction of *ACAN* mRNA expression in the cartilage of female rats normalized by *RPL4* + *HPRT1* at PW12 (*P* < 0.05; Figure 5H and Supplementary Figure 4D).

## Discussion

In this study, we used the three common gene expression stability analysis software to screen the optimal panel of reference genes (Al-Sabah et al., 2016; Lü et al., 2018; Sarker et al., 2018). The results showed that under physiological conditions, at the three stages of rat articular cartilage development (GD20, PW6, and PW12), the expression of *18S* was the highest, and the expression of *SDHA* was the lowest. All the three software showed that *RPL4, RPL5*, and *SDHA* had high stability, but there were specific differences, which may be related to the different algorithms used by each software. After comprehensively sorting the results of the three software, we found that *RPL4* and *RPL5* were the most stable genes at GD20, PW6, and PW12 in female rat cartilage and at GD20 and PW12 in male under physiological conditions. However, at PW6 in male rats cartilage, *RPL5* and *SDHA* had the highest stability, and *RPL4* ranked fourth, which was different from females. Although the reason for the gender difference in *RPL4* stability at PW6 is unknown, *RPL4* can still be used as a reference gene due to its *M* < 0.15. In addition, although the stability of *SDHA* and *ACTB* in male rat cartilage at PW6 ranked in the top three, the stability in other groups was worse, and the expression levels of *SDHA* was lower. Considering the stability of the reference genes and the simplicity and economy of the experiments, *SDHA* and *ACTB* were not the best reference genes.

It was worth noting that under physiological conditions, *SDHA* was quite stable in male and female rat cartilage at all development stages. The reason for that, maybe, the cartilage belongs to a hypoxic tissue whose metabolism process is mainly anaerobic respiration, but the succinate ubiquinone oxidoreductase encoded by the *SDHA* is the main component in the mitochondrial respiratory chain (Lane et al., 1977; Holzer et al., 2019; Zhao et al., 2019). However, in this study, the comprehensive stability of *RPL4* and *RPL5* is still higher than that of *SDHA*, which may be related to *RPL4* and *RPL5* as ribosomal protein-coding genes. A large number of studies have shown that the expression of various ribosomal protein genes in tissues is very stable, and it has been widely used as a reference gene for RT-qPCR in human, animal and plant (Al-Sabah et al., 2016; Mitra et al., 2016; Sadangi et al., 2017; Xiang et al., 2018). Ribosomal protein is a crucial component of the basic physiological process in the cell, which is the key to maintaining cell development and tissue homeostasis (Robledo et al., 2008; Perucho et al., 2014; Cedraz de Oliveira et al., 2017). Therefore, to maintain the basic physiological function of cells, the expression of ribosomal protein is often quite stable in various tissues and developmental stages. Wang et al. considered that ribosomal protein coding gene could replace the traditional reference gene as a commonly used candidate in RT-qPCR assay (Wan et al., 2011). *RPL4* and *RPL5* encode the component proteins of ribosomal 60S large subunit (Pillet et al., 2015; Hofman et al., 2017). Studies from Kolkova et al. (2013) and Brattelid et al. (2010) have shown that *RPL4* is the most stable reference gene in ovarian tumors and rat myocardium. Al-Sabah also pointed out that *RPL4* is also one of the most stable reference genes in articular cartilage under mechanical stress (Al-Sabah et al., 2016). At the same time, there are still a large number of studies showing that *RPL5* is the most stable reference gene in various tissues, such as murine cornea (Ren et al., 2010), right heart failure tissue from human (Li et al., 2017), breast muscle of chicken (Marciano et al., 2020) and broilers cartilage (Hul et al., 2020). The above results are consistent with our results, indicating that *RPL4* and *RPL5* have high stability for RT-qPCR assay in a variety of tissues, including cartilage in the development. Besides, in this study, geNorm calculated that V2/3 in all three periods in female and male rat cartilage was less than 0.15, which indicated that two reference genes could be selected as the optimal panel of reference genes. In summary, under physiological conditions, there was no significant difference in the optimal panel of reference genes in male and female rat cartilage between *in utero* (GD20) and postnatal (PW6, PW12). The expression levels of *RPL4* and *RPL5* were quite stable in the articular cartilage of male and female rats at all stages of development. They could be used as the optimal panel of reference genes.

To further verify the reliability of the selected reference genes in physiology, we detect the expression of the candidate reference genes in the cartilage development of male and female rats in the PDE-induced IUGR model which is a common experimental model to study cartilage development-derived diseases (Rowas et al., 2012; Tie et al., 2016; Xie et al., 2018; Cai et al., 2019). The results showed that *RPL4* and *RPL5* were also the optimal panel of reference genes in female rat cartilage at GD20 and PW6, which was consistent with physiological conditions; at PW12, geNorm also believed that *RPL4* and *RPL5* were the most stable reference genes in female rat cartilage, but NormFinder and Bestkeeper believed that *RPL5* and *ACTB* were the most stable reference genes, while *RPL4* was relatively unstable. The difference may be due to the different algorithms of the three software. Therefore, if only geNorm ranking was considered, *RPL4* and *RPL5* were still the optimal panel of reference genes in female rat cartilage at PW12. In the male rats of PDE model, RPL4 and RPL5 still had the highest stability at GD20 and PW12 and were the optimal panel of reference genes. However, at PW6, *RPL5* and *SDHA* had the highest stability, but *SDHA* expression was low, and *RPL4* ranked third. Also, considering the stability of the reference gene and the simplicity and economy of the experiments, *RPL4* and *RPL5* were still selected as the most proper panel of reference genes in the cartilage of male rats at PW6. In summary, consistent with physiological conditions, *RPL4* and *RPL5* were still the best panel of reference genes in the articular cartilage of male and female rats at developmental stages in the PDE model. It suggested that the reference gene selected in the cartilage at the developmental stages in physiology was still suitable for the PDE-induced IUGR rat model. Because fetal-maternal glucocorticoid overexposure may be a common mechanism of IUGR and multiple organ developmental disorders caused by the poor environment during pregnancy (Lesage et al., 2001; Chen et al., 2007; Liang et al., 2011; Buss et al., 2012; Pawluski et al., 2012; Xu et al., 2012; Cuffe et al., 2014; Correia-Branco et al., 2015; Sedaghat et al., 2015; He et al., 2017), the panel of reference genes selected under the PDE-induced rats IUGR model may also be applicable to rats IUGR models induced by other factors, but further verification is required.

Minimum Information for Publication of RT-qPCR Experiments proposes that a single reference gene to standardize the target genes will affect the accuracy of the results (Huggett et al., 2013). Therefore, to ensure the reliability and rigor of RT-qPCR analysis, it is of considerable significance to screen the stable panel of reference genes in rat developmental periods and verify their reliability. Previous studies showed that the matrix content of cartilage in each of the above periods decreased significantly in the PDE model, and the mRNA and protein expression levels of *ACAN* were also significantly reduced (Iwaniak et al., 2016; Chen et al., 2018; Xiao et al., 2020). To further verify the reliability of reference genes screened above, we used different reference genes to standardize the mRNA expression of *ACAN* in offspring rat cartilage in the PDE model. The results showed that the mRNA expression levels of *ACAN* were significantly reduced using *RPL4* and *RPL5* to normalize the mRNA expression. When using *GAPDH* and *ACTB* commonly used previously, the *ACAN* expression only decreased in some groups, indicating that their stability was not equal to *RPL4* and *RPL5*. By other single reference genes (*GAPDH, ACTB, 18S, GUSB*, and *HPRT1*), there were no significant differences. Further, using a panel of reference genes for normalization, the results showed that the expression levels of *ACAN* in male and female rat cartilage at various developmental stages were more considerably reduced when we used *RLP4* + *RPL5* than a single reference gene. And there was no obvious difference by other panel of reference genes (*GUSB* + *HPRT1*; *RPL4* + *HPRT1*), but they were better than their respective single reference genes. These results suggested that selecting the optimal panel of reference genes as a reference could reduce the differences within the group and was more conducive to obtaining a stable and accurate experimental conclusion.

## Conclusion

In summary, the present study screened and determined that the optimal panel of reference genes was *RPL4* + *RPL5* in the developmental period of male and female rats by detecting the expression of the candidate reference genes in the cartilage at different developmental stages (GD20, PW6, and PW12) in physiology and PDE-induced IUGR model. Furthermore, it was confirmed that there was no noticeable difference in gender and selection of the optimal panel of reference genes in the cartilage before and after birth. The present study provided a theoretical and experimental basis for further research on developmental cartilage diseases.

## Data Availability Statement

The raw data supporting the conclusions of this article will be made available by the authors, without undue reservation.

## Ethics Statement

The animal study was reviewed and approved by the Animal Experimental Ethics Committee of Wuhan University Medical College.

## Author Contributions

LL, QL, HH, MC, and SZ designed and performed the experiments. LC and HW analyzed the data. LL, HH, and HW were involved in writing the manuscript and approved the final manuscript. All authors contributed to the article and approved the submitted version.

## Conflict of Interest

The authors declare that the research was conducted in the absence of any commercial or financial relationships that could be construed as a potential conflict of interest.

## Abbreviations

*18S*: 18s ribosomal RNA
*ACAN*: aggrecan
*ACTB*: actin beta
C_*t*_: circulation threshold
CV: coefficient of variation
*GAPDH*: glyceraldehyde 3-phosphate dehydrogenase
GD: gestational day
*GUSB*: beta-glucuronidase
*HPRT1*: phosphoribosyltransferase 1
IUGR: intrauterine growth retardation
MIQE: Minimum Information for Publication of RT-qPCR
mRNA: messenger RNA
PDE: prenatal dexamethasone exposure
PW: postnatal week
*RPL4*: ribosomal protein L4
*RPL5*: ribosomal protein L5
RT-qPCR: real-time fluorescence quantitative polymerase chain reaction
SD: standard deviation
*SDHA*: complex flavoprotein subunit A.
