# Supplementary material for: Selection and Validation of the Optimal Panel of Reference Genes for RT-qPCR Analysis in the Developing Rat Cartilage
Source: Front Genet. 2020 Dec 16;11:590124. doi: 10.3389/fgene.2020.590124 (PMC7772434; doi:10.3389/fgene.2020.590124)
Supplement: Supplementary file 1 [file Data_Sheet_1.docx]

**Table S1．The full name, abbreviation, and function of reference genes.**

| **Full name** | **Abbreviation** | **Function** |
| --- | --- | --- |
| Glyceraldehyde-3-phosphate dehydrogenase | *GAPDH* | This gene encodes the key enzyme in glycolysis that widely distributes in various tissues and cells. |
| Actin beta | *ACTB* | This gene encodes one of the cytoskeletal actin. |
| 18s ribosomal RNA | *18S* | This gene encodes small ribosomal subunits that are widely found in animal and plant cells. |
| Hypoxanthine phosphoribosyltransferase 1 | *HPRT1* | The protein encoded by this gene plays a vital role in the purine salvage synthesis pathway. |
| Ribosomal protein L4 | *RPL4* | This gene encodes a protein of the ribosome, which is one of the components of the ribosome. |
| Succinate dehydrogenase  complex flavoprotein subunit A | *SDHA* | This gene encodes a major catalytic subunit of ubiquinone succinate oxidoreductase that is a component of the respiratory chain. |
| Glucuronidase beta | *GUSB* | This gene encodes a hydrolase that degrades glycosaminoglycans. |
| Ribosomal protein L5 | *RPL5* | This gene encodes a protein of the ribosome, which is one of the components of the ribosome. |

**Table S2．The primers used for a real-time quantitative polymerase chain reaction.**

| **Genes** | **Forward Primer** | **Reverse Primer** | **Annealing(℃)** |
| --- | --- | --- | --- |
| *GAPDH* | GGGTGTGAACCACGAGAAAT | ACTGTGGTCATGAGCCCTTC | 60 |
| *ACTB* | GGACCTGACAGACTACCTCA | GTTGCCAATAGTGATGACCT | 58 |
| *18S* | GTAAACCCGTTGAACCCCATT | CCATCCAATCGGTAGTAGCG | 59 |
| *HPRT1* | CTCATGGACTGATTATGGACAGG | AACAAAGTCTGGCCTGTATCC | 59 |
| *RPL4* | CCGGAAGTTGGATGAGTTGT | TTTGCGTAAGGGTTCAGCTT | 59 |
| *SDHA* | CGAGATCCGTGAAGGAAGAG | GCCCATGTTGTAATGCACAG | 58 |
| *GUSB* | CATGACGAACCAGTCACCAC | ACGGTCTGCTTCCCATACAC | 60 |
| *RPL5* | CCCGAACTACAACTGGCAAT | CATTCTGACCCATGATGTGC | 59 |
| *ACAN* | CTGGAAGAGAGGACACAGAAAC | CTCCTGAAGCTGAGGTCTCTA | 59 |

**Table S3．Candidate reference genes amplification efficiencies and standard curve parameters.**

| **Genes** | **Amplification efficiency(%)** | **R^2^** | **Standard curve slope** |
| --- | --- | --- | --- |
| *GAPDH* | 97 | 0.996 | -3.396 |
| *ACTB* | 101 | 0.999 | -3.298 |
| *18S* | 103 | 0.999 | -3.252 |
| *HPRT1* | 100 | 0.998 | -3.322 |
| *RPL4* | 98 | 0.997 | -3.371 |
| *SDHA* | 105 | 0.998 | -3.208 |
| *GUSB* | 99 | 0.997 | -3.346 |
| *RPL5* | 102 | 0.996 | -3.275 |


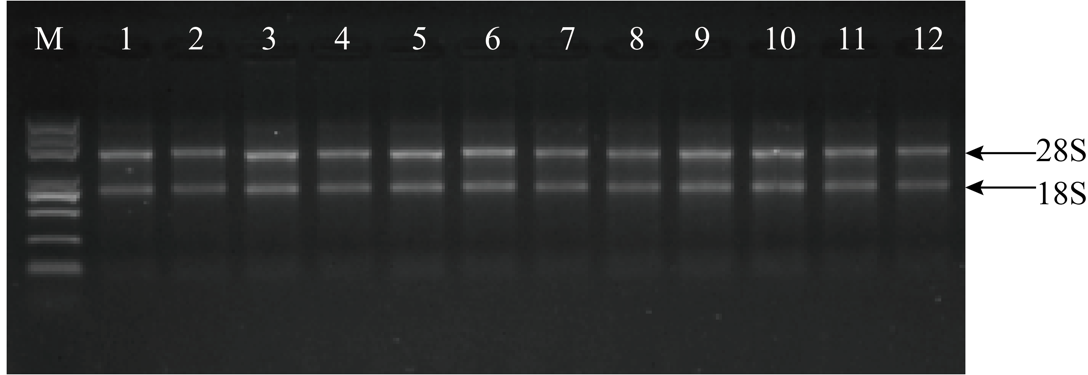


**Figure S1. The integrity of total RNA from rat cartilage.** M means marker; 1-12 represent the total RNA samples from these groups of GD20-Physiology-Female, GD20-Physiology-Male, PW6-Physiology-Female, PW6-Physiology-Male, PW12-Physiology-Female, PW12-Physiology-Male, GD20-PDE-Female，GD20-PDE-Male，PW6-PDE-Female，PW6-PDE-Male, PW12- PDE -Female and PW12-PDE-Male.


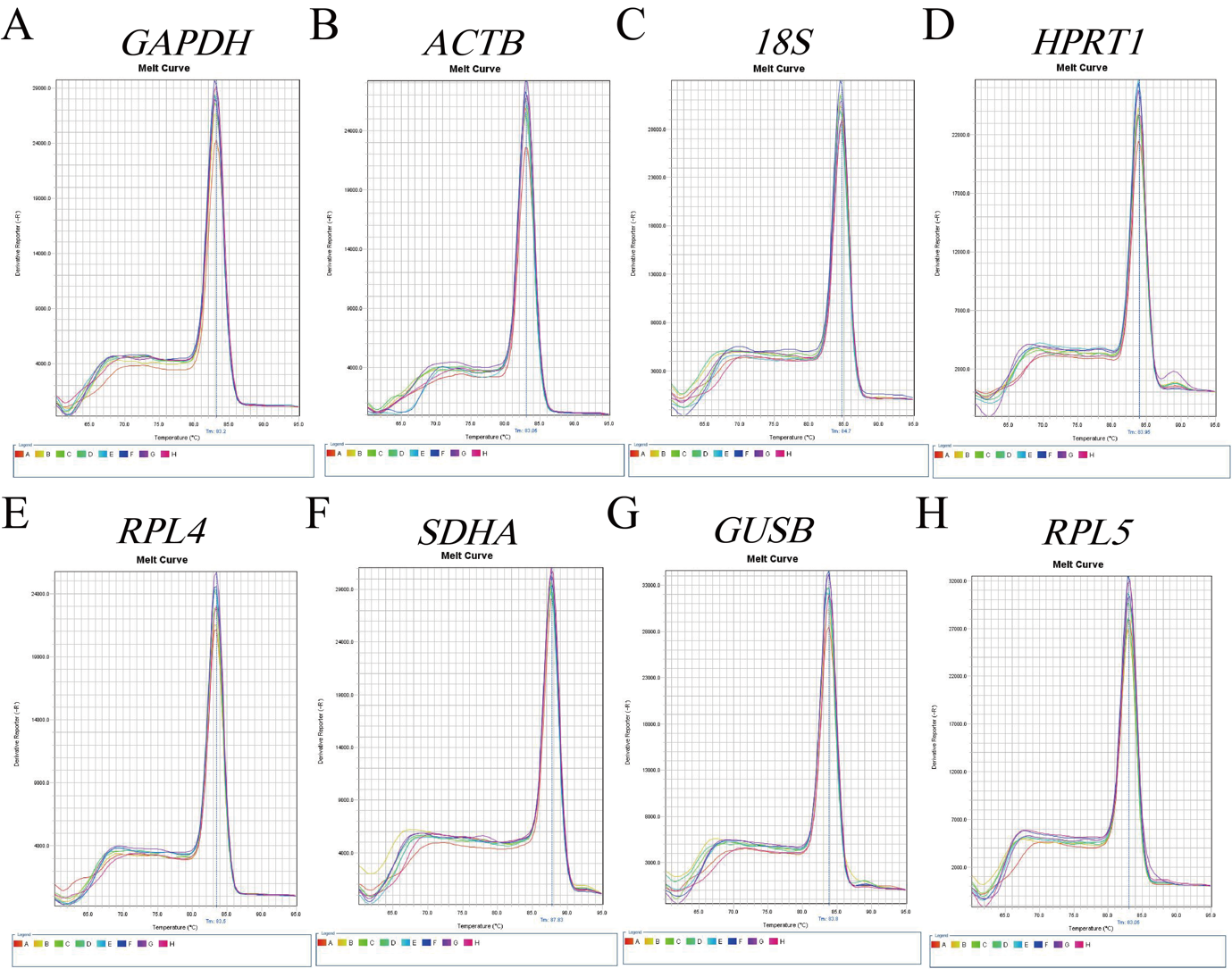


**Figure S2. Melting curve of candidate reference genes.** The melt curves of *GAPDH* (A), *ACTB* (B), *18S* (C), *HPRT1* (D), *RPL4* (E), *SDHA* (F), *GUSB* (G) and *RPL5*(H). n=8.


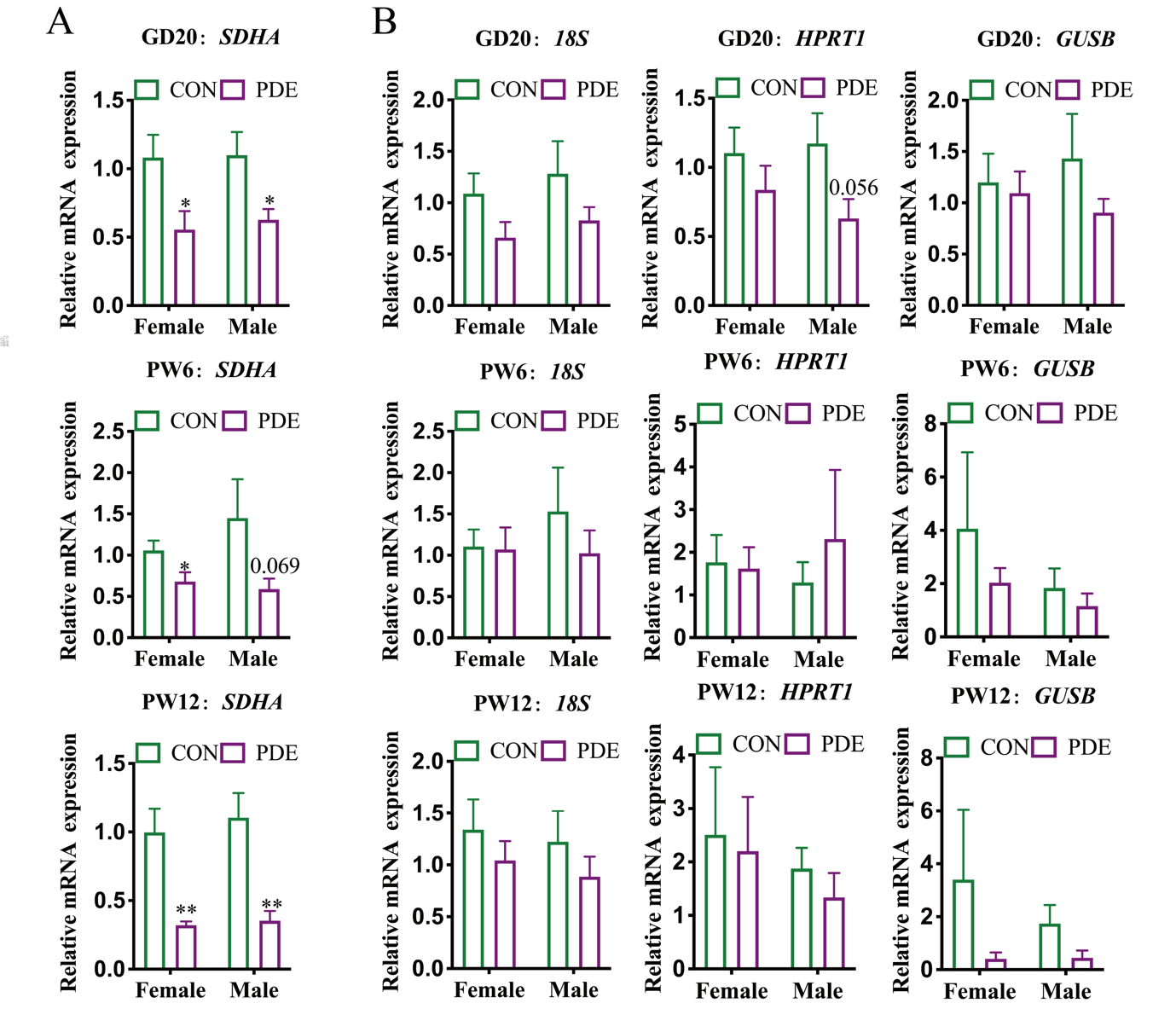


**Figure S3. Using single reference genes to count the effects of PDE on the expression of *ACAN* mRNA in rat cartilage.** (A-B) *ACAN* mRNA expression normalized by different single reference genes. The values were the means±S.E.M, n=8. ^*^*P*<0.05, ^**^*P*<0.01 *vs* control.


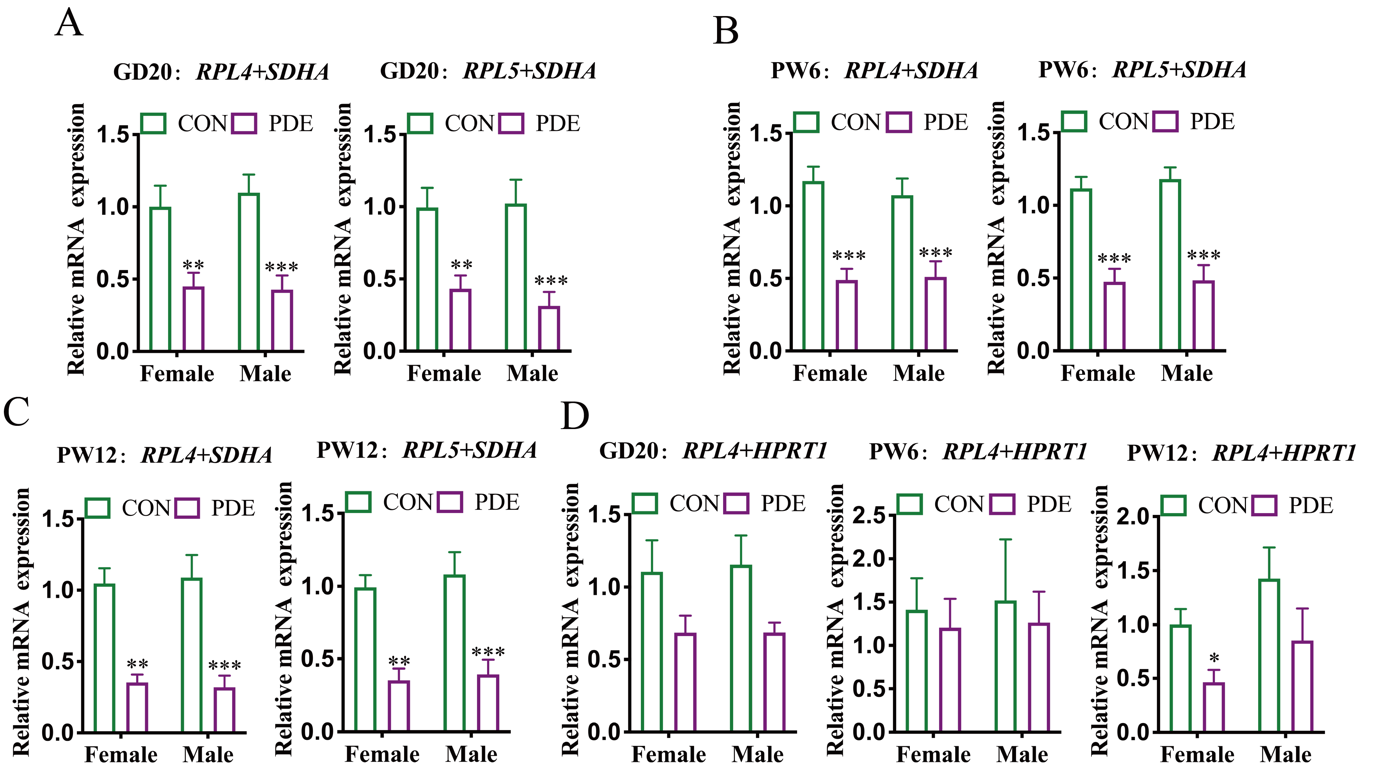


**Figure S4. Using compound reference genes to count the effects of PDE on the expression of *ACAN* mRNA in rat cartilage.** (A-D) *ACAN* mRNA expression normalized by different compound reference genes at GD20, PW6 and PW12. The values were the means ± S.E.M, n=8. ^*^*P*<0.05, ^**^*P*<0.01, ^***^*P* <0.001 *vs* control.
